# Prevalence and associated knowledge of hepatitis B infection among healthcare workers in Freetown, Sierra Leone

**DOI:** 10.1186/s12879-018-3235-1

**Published:** 2018-07-09

**Authors:** Yu-Ling Qin, Bo Li, Yue-Su Zhou, Xin Zhang, Lei Li, Bing Song, Peng Liu, Yue Yuan, Zhong-Peng Zhao, Jun Jiao, Jing Li, Yi Sun, Stephen Sevalie, Joseph E. Kanu, Ya-Jun Song, Jia-Fu Jiang, Foday Sahr, Tian-Jun Jiang, Chinese Military Medical Experts Group in Sierra Leone, Yu-Ling Qin, Bo Li, Yue Yuan, Yi Sun, Jing Li, Zhong-Peng Zhao, Jun Jiao, Ya-Jun Song, Tian-Jun Jiang, Jia-Fu Jiang

**Affiliations:** 1 Beijing 302 Hospital, Beijing, China; 2 Beijing Institute of Microbiology and Epidemiology, Beijing, China; 3 No. 34 Military Hospital, Wilberforce, Freetown, Sierra Leone; 4 College of Medicine and Allied Health Sciences, Freetown, Sierra Leone

**Keywords:** Hepatitis B virus, Healthcare workers, Prevalence, Sierra Leone

## Abstract

**Background:**

Hepatitis B virus (HBV) is considered highly prevalent in West Africa. However, major gaps in surveillance exist in Sierra Leone. Although healthcare workers (HCWs) are at high risk for HBV infection, little is known about the prevalence and knowledge of hepatitis B among HCWs in Sierra Leone.

**Methods:**

A cross-sectional study of all HCWs at the No. 34 Military Hospital located in Freetown, Sierra Leone, was conducted from March 20 to April 10, 2017. Whole blood was collected and screened for HBV markers using a one-step rapid immunochromatographic test with positive samples tested for HBV DNA. Additionally, questionnaires assessing self-reported knowledge of HBV infections were administered to all participants. Data were processed and analyzed using SPSS (version 17.0) software.

**Results:**

A total of 211 HCWs were included in this study with a median age of 39.0 years (range: 18–59). Of the participating HCWs, 172 (81.5%) participants were susceptible (all markers negative), 21(10.0%) were current HBV (HBsAg positive) and nine (4.3%) were considered immune because of past infection (HBsAg negative and anti-HBc positive; anti-HBs positive). Additionally, nine (4.3%) participants displayed immunity to the virus as a result of prior hepatitis B vaccination (only anti-HBs positive). Of the 21 HCWs with positive HBsAg, 13 (61.9%) had detectable HBV DNA. There was a significantly lower risk for current HBV infection among HCWs older than 39 years (OR 0.337, *p* = 0.046). In addition, only 14 (6.6%), 73 (34.6%) and 82 (38.9%) participants in this survey had adequate knowledge about the clinical outcome, routes of transmission, and correct preventive measures of HBV infection, respectively.

**Conclusions:**

HCWs in Sierra Leone lacked adequate knowledge of the hepatitis B virus. Additionally, the low coverage rate of hepatitis B vaccination among HCWs fails to meet WHO recommendations, leaving many of the sampled HCWs susceptible to infection. This study reaffirms the need for more intensive training for HCWs in addition to strengthening vaccination programmes to protect HCWs against HBV in Sierra Leone.

**Electronic supplementary material:**

The online version of this article (10.1186/s12879-018-3235-1) contains supplementary material, which is available to authorized users.

## Background

Hepatitis B virus (HBV) infection is a major threat to public health globally. According to the World Health Organization (WHO), approximately 257 million people are infected with chronic HBV [1]. Infection with HBV can increase the risk of death from liver cirrhosis and hepatocellular carcinoma, which is the sixth most common cancer and the third cause of cancer death worldwide [2]. The WHO reported that hepatitis B prevalence is the highest in the western Pacific region and Africa, where 6.2 and 6.1% of the adult population are infected, respectively [1]. Sierra Leone, located in West Africa, is considered a high-endemic area. A study conducted among primary school children, in 1998, detected HBsAg in 18% of the children tested [3]. In 2005, a study showed a 6.2% seroprevalence of hepatitis B among pregnant women of middle and high socio-economic class in Sierra Leone [4]. Recently, a study screening blood donor candidates for blood-borne pathogens found a HBsAg prevalence of 15% in men and 13% in women from a single hospital in Tonkolili Province, Sierra Leone [5]. These results illustrate the serious public health risks that hepatitis B poses in Sierra Leone. In addition, HBV continues to be an understudied topic in Sierra Leone. Due to limited resources, little has been done to combat hepatitis B in Sierra Leone despite the suspected high burden of disease. Although the hepatitis B vaccine was introduced for 6 to 14 week-old children, there are currently no active programs administering the vaccine at birth. Furthermore no coordinated HBV vaccination program been put in place to prevent infection among the adult population [6].

Healthcare workers (HCWs) are considered a high-risk group for HBV infection due to occupational exposure to blood-borne pathogens. Previous studies in Africa found high HBV infection and exposure rates (roughly 10%) in HCWs in South Africa and Nigeria. Worldwide, approximately 2 million HCWs are infected with HBV through sharp injury [7–9]. This study was designed to evaluate the prevalence of HBV markers in HCWs as well as to assess their knowledge of HBV infection and prevention in Sierra Leone.

## Methods

### Study area and sample population

A cross-sectional study was conducted at the No. 34 Military Hospital in Freetown, Sierra Leone from March 20 to April 10, 2017. This hospital serves as a medical center for the Republic of Sierra Leone Armed Forces in addition to also being a teaching and general hospital. A total of HCWs, including medical doctors, nurses and other non-clinical health workers, were studied and written, informed consent was obtained from each subject.

### Laboratory detection of hepatitis B virus

Blood samples were drawn from the antecubital vein of the 211 participants by phlebotomists of the No. 34 Military Hospital clinical laboratory then centrifuged for 5 min at 12,000 g at room temperature. Serological tests were performed to detect five markers including HBsAg, anti-HBs, HBeAg, anti-HBe and anti-HBc, by using a one-step rapid immunochromatographic test (Shanghai Kehua Bio-engineering Co., Ltd., Shanghai, China). Test results were interpreted and reported as positive or negative based on the manufacturer’s instructions.

Viral deoxyribonucleic acid (DNA) was extracted using the QIAamp DNA Blood Mini Kit (QIAGEN, Germantown, MD, USA) according to the manufacturer’s instructions. HBV DNA was amplified using real-time PCR (qPCR) (Light Cycler Software Version 4.1, Roche Diagnostics, Penzburg, Germany) HBV DNA assay kits (Sansure Biotech, Changsha, China) in all HBsAg positive samples.

### Design and administration of the questionnaire

Data were collected using a self-administered questionnaire, which was developed after reviewing relevant research [10–12]. The survey had questions on socio-demographic characteristics, knowledge of HBV clinical outcome, route of transmission and preventive measures against hepatitis B infection, previous infection, and previous vaccination history. No vaccinations were offered to HCWs as part of this study. Seventeen questions had binary (yes or no) responses, and three multi-items questions focusing on the clinical outcome of HBV infection, route of transmission and proper preventive measures had only one correct answer. Each study participant was expected to complete the questionnaire.

### Statistical analysis

The data obtained from the questionnaire and the results of the laboratory test were analyzed using SPSS (version 17.0, SPSS Inc. Chicago, IL). The response for each question was given a score of one and zero indicating a right or wrong answer was provided, respectively. Then we summed and graded the total scores as ‘poor’, ‘intermediate’ or ‘adequate’ level for each study participant based on the distribution located in the tri-sectional quantiles of the grouped data array. The Pearson Chi-square test was used to determine the relationships between participant characteristics and HBV marker detection rates. Multivariable factor analysis for current HBV infection was carried out for seven possible risk factors, including age, gender, working experience years, education background, occupation, vaccination, and sharp injury history. Differences were considered statistically significant when the *p* value was < 0.05.

## Results

### Demographic characteristics among HCWs

The median age of the 211 HCWs who participated in the study was 39.0 years (range:18–59). Roughly half of participants were male (51.2%), over the age of 39 (46.9%), and had spent less than 9 years in their current job (46.4%). The majority of HCWs had a middle education level (Diploma Certificate, 78.7%) (Table 1).

**Table 1:** Prevalence of five hepatitis B virus serological markers by socio-demographic characteristics of the study participants

Characteristic	Tested no. (%)	HBsAg (+) no. (%)	*P* value	HBsAb (+) no. (%)	*P* value	HBeAg (+) no. (%)	*P* value	HBeAb (+) no. (%)	*P* value	HBcAb (+) no. (%)	*P* value
Age (y)											
≥ 39y	112 (53.1)	6 (5.4)	0.021^*^	7 (6.3)	0.342	0 (0.0)	0.469	7 (6.3)	0.027^*^	10 (8.9)	0.066
< 39y	99 (46.9)	15 (15.2)		3 (3.0)		1 (1.0)		16 (16.2)		18 (18.2)	
Gender											
Male	108 (51.2)	10 (9.3)	0.820	7 (6.5)	0.333	0 (0.0)	0.488	11 (10.2)	0.826	13 (12.0)	0.686
Female	103 (48.8)	11 (10.7)		3 (2.9)		1 (1.0)		12 (11.7)		15 (14.6)	
Working experience											
≥ 9y	113 (53.6)	8 (7.1)	0.168	7 (6.2)	0.345	0 (0.0)	0.464	8 (7.1)	0.076	10 (8.8)	0.066
< 9y	98 (46.4)	13 (13.3)		3 (3.1)		1 (1.0)		15 (15.3)		18 (18.4)	
Education level											
High school	39 (18.5)	6 (15.4)	0.365	1 (2.6)	0.645	0 (0.0)	0.873	5 (12.8)	0.807	7 (17.9)	0.601
Diploma certificate	166 (78.7)	14 (8.4)		9 (5.4)		1 (0.6)		17 (10.2)		20 (12.0)	
Bachelor’s degree or higher	6 (2.8)	1 (16.7)		0 (0.0)		0 (0.0)		1 (16.7)		1 (16.7)	
Occupation											
Medical doctor	7 (3.3)	2 (28.5)		0 (0.0)		0 (0.0)		3 (42.9)		3 (42.9)	
Nurse	169 (80.1)	17 (10.1)	0.182	9 (5.3)	0.687	1 (0.6)	0.883	18 (10.7)	0.015^*^	23 (13.6)	0.029^*^
Others staff	35 (16.6)	2 (5.7)		1 (2.9)		0 (0.0)		2 (5.7)		2 (5.7)	
Department											
Internal Medicine	21 (10.0)	1 (4.8)	0.560	1 (4.8)	0.071	0 (0.0)	0.338	2 (9.5)	0.637	2 (9.5)	0.384
Surgical Department	47 (22.3)	8 (17.0)		1 (2.1)		0 (0.0)		8 (17.0)		9 (19.1)	
Emergency Department	16 (7.6)	1 (6.3)		1 (6.3)		0 (0.0)		1 (6.3)		1 (6.3)	
Paediatric	19 (9.0)	2 (10.5)		0 (0.0)		1 (5.3)		2 (10.5)		4 (21.1)	
Obstetrics and Gynecology	9 (4.3)	0 (0.0)		0 (0.0)		0 (0.0)		0 (0.0)		0 (0.0)	
Under Fives Clinic	6 (2.8)	0 (0.0)		1 (16.7)		0 (0.0)		0 (0.0)		0 (0.0)	
Laboratory	16 (7.6)	0 (0.0)		0 (0.0)		0 (0.0)		0 (0.0)		0 (0.0)	
OP Theatre	22 (10.4)	2 (9.0)		1 (4.5)		0 (0.0)		2 (9.0)		2 (9.0)	
Pharmacy	5 (2.4)	1 (20.0)		2 (40.0)		0 (0.0)		1 (20.0)		1 (20.0)	
Others	50 (23.7)	6 (12.0)		3 (6.0)		0 (0.0)		7 (14.0)		9 (18.0)	
Hepatitis history											
YES	14 (6.6)	2 (14.3)	0.268	1 (7.1)	0.908	0 (0.0)	0.905	2 (14.3)	0.391	3 (21.4)	0.429
NO	176 (83.4)	15 (8.5)		8 (4.5)		1 (0.6)		17 (9.7)		21 (11.9)	
Unknown	21 (10.0)	4 (19.0)		1 (4.8)		0 (0.0)		4 (19.0)		4 (19.0)	
HBV vaccination											
YES	37 (17.5)	1 (2.7)	0.135	6 (16.2)	0.002^*^	0 (0.0)	1.000	1 (2.7)	0.087	1 (2.7)	0.034^*^
NO	174 (82.5)	20 (11.5)		4 (2.3)		1 (0.6)		22 (12.6)		27 (15.5)	
Sharps injury											
Never	110 (52.1)	11 (10.0)	0.955	5 (4.5)	0.739	1 (1.0)	0.630	14 (12.7)	0.586	15 (13.6)	0.962
Once	36 (17.1)	4 (11.1)		1 (2.8)		0 (0.0)		4 (11.1)		5 (13.9)	
More than once	65 (30.8)	6 (9.2)		4 (6.2)		0 (0.0)		5 (7.7)		8 (12.3)	
Total	211 (100)	21 (10.0)		10 (4.7)		1 (0.5)		23 (10.9)		28 (13.3)	

^*^Statistically significant at *P* < 0.05

### Prevalence of HBV

Of the 211 HCWs, the positive detection rates of the five markers HBsAg, anti-HBs, HBeAg, anti-HBe and anti-HBc were 10.0, 4.7, 0.5, 10.9 and 13.3%, respectively (Table 1). Twenty-one of the (10.0%) HCWs tested positive for current HBV infections (HBsAg positive, anti-HBc positive) (Table 2), nine (4.3%) were considered immune due to past infection (HBsAg negative and anti-HBc positive; anti-HBs positive), and nine (4.3%) participants were immune due to hepatitis B vaccination (only anti-HBs positive). In total, 172 (81.5%) participants were considered susceptible (all markers negative) (Table 2). Among 21 participants who were HBsAg positive, 13 (61.9%) were HBV DNA positive. Twelve of them were determined very low-level HBV DNA (< 10^3) and one had a DNA concentration of 5.6 × 10^3 copies/mL.

**Table 2:** Summary of hepatitis B virus infection status among HCWs in the hospital, Sierra Leone

HBV infection classification	Number (%) (*n* = 211)
Susceptible	172 (81.5)
All markers negative	172 (81.5)
Acute or chronic infection	21 (10.0)
HBsAg (+), HBeAb (+), HBcAb (+)	19 (9.0)
HBsAg (+), HBeAb (+)	1 (0.5)
HBsAg (+), HBcAb (+)	1 (0.5)
Immune due to hepatitis B vaccination	9 (4.0)
Only HBsAb (+)	9 (4.3)
Immune due to natural infection	9 (4.0)
Only HBcAb (+)	5 (2.4)
HBeAb (+), HBcAb (+)	2 (0.9)
HBsAb (+), HBeAb (+)	1 (0.5)
HBeAg (+), HBcAb (+)	1 (0.5)

Anti-HBs positive rate of participants who reported receiving a HB vaccine significantly increased. This was in comparison to participants who had not received vaccination (16.2% vs. 1.7%, *p* = 0.001) (Table 2). Of the 211 participants, only 14 (6.6%) participants had clear HBV infection history, out of which 4 tested positive for serological markers (Table 2). The prevalence of the “current infection” group was significantly higher in HCWs < 39 years old (*p* = 0.018) (Table 3). Multivariable factor analysis for risk for current HBV infection showed that there was a significantly lower risk for current HBV infection among those HCWs aged > 39 years (OR = 0.337; 95% CI:0.116–0.980; *p* = 0.046) (Table 4).

**Table 3:** Prevalence of four hepatitis B virus infection status by socio-demographic characteristics of the study participants

Characteristic	Tested no. (%)	Susceptible no. (%)	*P* value	Current infection no. (%)	*P* value	Past infection no. (%)	*P* value	Immune due to vaccination no. (%)	*P* value
Age (y)			0.337		0.018^*^		0.506		0.506
≥ 39y	112 (53.1)	94 (83.9)		6 (5.4)		6 (5.4)		6 (5.4)	
< 39y	99 (46.9)	78 (78.8)		15 (15.2)		3 (3.0)		3 (3.0)	
Gender			0.989		0.730		0.744		0.499
Male	108 (51.2)	88 (81.5)		10 (9.3)		4 (3.7)		6 (5.6)	
Female	103 (48.8)	84 (81.6)		11 (10.7)		5 (4.9)		3 (2.9)	
Working experience			0.452		0.419		0.509		0.736
≥ 9y	113 (53.6)	90 (79.6)		13 (11.5)		6 (5.3)		4 (3.5)	
< 9y	98 (46.4)	82 (83.7)		8 (8.2)		3 (3.1)		5 (5.1)	
Education level			0.933		0.365		0.716		0.716
High school	39 (18.5)	31 (79.5)		6 (15.4)		1 (2.6)		1 (2.6)	
Diploma certificate	166 (78.7)	136 (81.9)		14 (8.4)		8 (4.8)		8 (4.8)	
Bachelor’s degree or higher	6 (2.8)	5 (83.3)		1 (16.7)		0 (0.0)		0 (0.0)	
Occupation			0.736		0.894		0.311		0.780
Medical doctor	7 (3.3)	6 (85.7)		1 (14.3)		0 (0.0)		0 (0.0)	
Nurse	169 (80.1)	136 (80.5)		17 (10.1)		9 (5.3)		7 (4.1)	
Other staff	35 (16.6)	30 (85.7)		3 (8.6)		0 (0.0)		2 (5.7)	
Department			0.000^*^		0.560		0.738		0.007^*^
Internal medicine	21 (10.0)	18 (85.7)		1 (4.8)		1 (4.8)		1 (4.8)	
Surgical Department	47 (22.3)	36 (76.6)		8 (17.0)		3 (6.4)		0 (0.0)	
Emergency Department	16 (7.6)	14 (87.5)		1 (6.3)		0 (0.0)		1 (6.3)	
Pediatric	19 (9.0)	15 (78.9)		2 (10.5)		2 (10.5)		0 (0.0)	
Obstetrics and Gynecology	9 (4.3)	0 (0.0)		0 (0.0)		0 (0.0)		0 (0.0)	
Under Fives Clinic	6 (2.8)	5 (83.3)		0 (0.0)		0 (0.0)		1 (16.7)	
Laboratory	16 (7.6)	16 (100.0)		0 (0.0)		0 (0.0)		0 (0.0)	
OP Theatre	22 (10.4)	19 (40.9)		2 (9.0)		0 (0.0)		1 (4.5)	
Pharmacy	5 (2.4)	2 (40.0)		1 (20.0)		0 (0.0)		2 (40.0)	
Others	50 (23.7)	38 (76.0)		6 (12.0)		3 (6.0)		3 (6.0)	
Hepatitis history			0.355		0.854		0.110		0.393
YES	14 (6.6)	10 (71.4)		2 (14.3)		2 (14.3)		0 (0.0)	
NO	176 (83.4)	143 (81.3)		17 (9.7)		7 (4.0)		9 (5.1)	
Unknown	21 (10.0)	19 (90.5)		2 (9.5)		0 (0.0)		0 (0.0)	
HBV vaccination			0.588		0.135		1.000		0.001^*^
YES	37 (17.5)	29 (78.4)		1 (2.7)		1 (2.7)		6 (16.2)	
NO	174 (82.5)	143 (82.2)		20 (11.5)		8 (4.6)		3 (1.7)	
Sharps injury			0.025^*^		0.351		0.670		0.063
Never	110 (52.1)	82 (74.5)		14 (12.7)		6 (5.5)		8 (7.3)	
Once	36 (17.1)	32 (88.9)		2 (5.6)		1 (2.8)		1 (2.8)	
More than once	65 (30.8)	58 (89.2)		5 (7.7)		2 (3.1)		0 (0.0)	
Total	211 (100)	172 (81.5)		21 (10.0)		9 (4.3)		9 (4.3)	

^*^Statistically significant at *P* < 0.05

**Table 4:** Multivariable analysis of possible risk factors for current HBV infection

Variables	Category	Frequency	OR	95% CI for OR	*P* Value
Age	≥39y	6/112	0.337	0.116–0.980	0.046^*^
	<39y	15/99			
Gender	Male	10/108	1.304	0.471–3.609	0.609
	Female	11/103			
Working experience	≥9y	13/113	1.334	0.502–3.547	0.563
	<9y	8/98			
Education background	Low	6/39	0.401	0.104–1.547	0.185
	Mid	14/166			
	High	1/6			
Occupation	Doctors	1/7	2.114	0.496–9.018	0.312
	Nurses	17/169			
	Others	3/35			
Vaccination	Yes	1/37	0.291	0.036–2.383	0.250
	No	20/174			
Sharps injury	Never	14/110	0.692	0.391–1.225	0.207
	Once	2/36			
	More than once	5/65			

^*^Statistically significant at *P* < 0.05

### Knowledge of HBV infection and associated factors

According to participants’ responses, 77.3% (163/211) of staff were not aware of clinical outcomes of HBV infection, while 63 (29.9%) and 93(44.1%) had a poor knowledge on transmission routes and preventive measures of HBV, respectively. The survey also revealed that working experience was associated with greater knowledge of preventive measures for HBV(*p* = 0.017) and medical doctors were more knowledgeable about the consequences of HBV infection (*p* = 0.05) (Table 5).

**Table 5:** Responses of the study participants to basic hepatitis B knowledge

Characteristic	The consequences of infection				Route of transmission				Preventive measures			
	no. (%)			*P* value	no. (%)			*P* value	no. (%)			*P* value
	Poor	Intermed	Good		Poor	Intermed	Good		Poor	Intermed	Good	
Age (y)												
< 39y	77 (77.8)	15 (15.2)	7 (7.1)	0.920	31 (31.3)	35 (35.4)	33 (33.3)	0.896	41 (41.4)	17 (17.2)	41 (41.4)	0.736
≥ 39y	86 (76.8)	19 (16.9)	7 (6.3)		32 (28.6)	40 (35.7)	40 (35.7)		52 (46.4)	19 (16.9)	41 (36.7)	
Gender												
Female	75 (72.8)	20 (19.4)	8 (7.8)	0.322	26 (25.4)	42 (40.8)	35 (33.9)	0.222	42 (41.7)	19 (18.5)	42 (40.8)	0.634
Male	88 (81.5)	14 (12.9)	6 (5.6)		37 (34.3)	33 (30.6)	38 (35.2)		51 (47.2)	17 (15.7)	40 (37.0)	
Working experience												
< 9y	74 (75.5)	19 (19.4)	5 (5.1)	0.380	27 (27.6)	35 (35.7)	36 (36.7)	0.752	33 (33.7)	19 (19.4)	46 (46.9)	0.017^*^
≥ 9y	89 (78.8)	15 (13.3)	9 (7.9)		36 (31.9)	40 (35.4)	37 (32.7)		60 (53.1)	17 (15.0)	36 (31.9)	
Education level												
High school	25 (65.8)	9 (23.7)	4 (10.5)	0.324	9 (20.5)	16 (41.0)	13 (33.3)	0.378	15 (38.5)	9 (23.1)	14 (35.9)	0.324
Diploma certificate	133 (79.6)	24 (14.4)	10 (5.9)		51 (31.7)	58 (35.4)	58 (35.4)		75 (45.7)	26 (15.9)	66 (40.2)	
Bachelor’s degree or higher	5 (83.3)	1 (16.7)	0 (0.0)		3 (37.5)	1 (12.5)	2 (25.0)		3 (50.0)	1 (16.7)	2 (33.3)	
Occupation												
Medical doctor	4 (57.1)	2 (28.6)	1 (14.3)	0.050^*^	0 (0.0)	3 (42.9)	4 (57.1)	0.196	2 (28.6)	1 (14.3)	4 (57.1)	0.221
Nurse	137 (81.1)	21 (12.4)	11 (6.5)		56 (33.1)	59 (34.9)	54 (31.9)		80 (47.3)	30 (17.8)	59 (34.9)	
Other staff	22 (62.9)	11 (31.4)	2 (5.7)		7 (20.0)	13 (37.1)	15 (42.9)		11 (31.4)	5 (14.3)	19 (54.3)	
Total	163 (77.3)	34 (16.1)	14 (6.6)		63 (29.9)	75 (35.5)	73 (34.6)		93 (44.1)	36 (17.1)	82 (38.9)	

^*^Statistically significant at *P* < 0.05

## Discussion

The global prevalence of hepatitis B is among the highest in parts of Africa, containing an estimated 50 million chronic carriers of HBV [13, 14]. Previous studies have shown HBsAg positive rates above 10% in African countries such as Burkina Faso, the Central African Republic, and Nigeria [15–17]. While in Sierra Leone reported HBsAg prevalence varied with different populations and times. Reports indicated a prevalence of 18% among children at a primary school in capital in 1998 [3], 6.2% among pregnant women of middle and high socio-economic status in 2005 [4], and 13–15% among blood donor candidates in Tonkolili District in 2017 [5]. In our present study, the sero-prevalence of HBsAg in HCWs was 10.0%, which is similar to rates observed in Uganda [18], but higher than those in Nigeria (1.5%) [19] and drastically higher than the developed European Region [20]. However, our study also found that the prevalence of anti-HBs was only 4.7% as compared to a South African report which detected a 19.9% anti-HBs positive rate in HCWs [21]. These results, especially the high prevalence of HBsAg and current HBV infection, suggest that hepatitis B is a very serious health concern in Sierra Leone. Our study also showed that the HBeAg positivity was low, but HBV DNA positivity was quite high (62%). This may be indicative of the effects of HBV pre-C mutation in the study population, another issues which requires further investigation.

A significant difference in current infection rate between participants younger than 39 years old (*p* = 0.018) was observed in this study. Additionally, multivariable analysis of possible risk factors suggests a lower risk for current HBV infection among those HCWs aged > 39 years (OR = 0.337; 95% CI:0.116–0.980; *p* = 0.046). This finding may be due to differences in lifestyle or behavior between the two groups, however a larger sample size is needed to adequately study this risk factor. As expected, we also found that HBV vaccination was a protective factor for anti-HBs positive (immune status due to vaccination), as demonstrated elsewhere [22]. Before the vaccination program was launched in 1995, HBsAg carriage in the African population was very high [23]. In Sierra Leone, the hepatitis B vaccine is not available for the entire population because of limited resources. Our study found that only 17.5% HCWs reported previous HBV vaccination history, which is higher than those in the Democratic Republic of Congo (3.6%) [10] and Ethiopia (5.4%) [24]. However, only 16.2% of those vaccinated produced protective antibodies against HBV. This may have resulted from receiving a vaccination many years ago thereby resulting in waning immunity. In addition, there were three individuals who did not report previous HBV vaccination, but had similar immunological results to those who had been vaccinated, which was likely a result of recall bias. Vaccination of HCWs for HBV has been recommended by the WHO. However, even in South Africa, where there is a stronger healthcare system, only 30.6–52.4% of HCWs had protective levels of anti-HBs [7]. In addition, 81.5% HCWs in Sierra Leone tested negative for all markers, indicating susceptibility to HBV infection. Thus, there is an urgent need to expand vaccination coverage rates among HCWs in Sierra Leone.

The present study also found that there was poor knowledge of HBV, including the clinical outcome of infection, route of transmission and preventive measures of HBV, among HCWs. Therefore, HCWs in Sierra Leone will continue to be at risk of HBV infections until training and vaccination programs are strengthened. Our study also demonstrates that HCWs with longer working experience had more knowledge about preventive measures as compared to those with less work experience (*p* = 0.007). Furthermore, medical doctors had more knowledge of the consequences of HBV infection (*p* = 0.05), as expected compared to other occupations. It is generally assumed that education level and departments correlate with overall knowledge levels of the infection. However, this was not the case as these factors were not significant across all categories of knowledge, which is likely due to the small sample size of specific departments and education levels.

### Limitations

The data presented in this study comes from a single hospital, which may not be representative of other healthcare facilities in Sierra Leone. It would be premature to draw broader conclusions regarding the prevalence and knowledge of hepatitis B across all HCWs in Sierra Leone.

## Conclusions

HCWs in Sierra Leone lacked adequate knowledge of the hepatitis B virus.. Additionally, there were low coverage rates of hepatitis B vaccination that does not seem to be able to meet the WHO recommendations, leaving many HCWs susceptible to hepatitis B infection in the sampled population. Local health authorities need to make a coordinated effort to increase vaccination uptake considering the cost-effectiveness of broad immunization against hepatitis B and incorporate more intensive training against blood-borne pathogens for HCWs in Sierra Leone.

## Additional file

Additional file 1: The original data of HBV surveillance for HCWs from Sierra Leone. (XLSX 25 kb)

## Abbreviations

DNA: Deoxyribonucleic acid
HBV: Hepatitis B virus
HCWs: Healthcare workers
WHO: World Health Organization
